# Would you do it again? A qualitative study of student and supervisor perceptions of an intercalated MBChB/PhD programme

**DOI:** 10.1186/s12909-019-1909-z

**Published:** 2019-12-26

**Authors:** Yassar Alamri, Kate Magner, Tim J. Wilkinson

**Affiliations:** 1 New Zealand Brain Research Institute, Christchurch, New Zealand; 2 Department of Medicine, Christchurch Public Hospital, Christchurch, New Zealand; 3 Canterbury District Health Board, 2 Riccarton Avenue, Christchurch, 8011 New Zealand; 4 Medical Education Unit, University of Otago, Christchurch, New Zealand

**Keywords:** Physician-scientist, Intercalated degree, Medical student, Research, New Zealand

## Abstract

**Background:**

Several studies have warned about the diminishing physician-scientist breed. Limited studies, however, have attempted to assess what factors (if any) enhanced or hindered the experience of trainee physician-scientists and their supervisors. Using Vroom’s expectancy theory as a conceptual framework, we explored the highlights, motivations and barriers of an intercalated MBChB/PhD programme as experienced by students of the programme and their supervisors.

**Methods:**

Previous and current students of the MBChB/PhD programme at the University of Otago, and their supervisors, were invited to provide comments on the programme. Data were analysed using a general inductive approach which involved coding responses, and grouping codes into common themes via an iterative process. A deductive approach was used to interpret the themes and relate them to Vroom’s expectancy theory.

**Results:**

A total of 22 students (88% response rate) and 36 supervisors (58.3% response rate) responded to our survey. Three themes were identified through the analysis of the students’ responses. These were: motives for undertaking the intercalated degree, effect on career development, and perceived barriers. Supervisors’ survey yielded two themes: characteristics of successful students, and optimising the intercalated programme.

**Conclusions:**

The current study sheds light on the successes and challenges of an intercalated MBChB/PhD programme by considering the views of those most involved. Whereas the combined programme has its advantages for student research and career development, extending the research-time may be worthwhile. Further studies involving a larger cohort of intercalating students and their supervisors may allow for extrapolation of data to address these concerns.

## Introduction

There is no shortage of articles in the medical education literature lamenting the dwindling interest in physician-scientist training, and forewarning its adverse consequences [1, 2]. Limited studies, however, have attempted to assess what factors (if any) enhanced or hindered the experience of trainee physician-scientists and their supervisors. Kwan et al surveyed (using a multi-choice–based questionnaire) students from five academic medical schools in the US. For MD/PhD students, obstacles in balancing academic (clinical, research and educational) and family responsibilities were major considerations with regards to future career choices and research involvement [3]. There is a current knowledge gap in the experiences of medical/PhD students outside the US, as well as the views on the intercalated programme by senior academics supervising these students.

Currently, one of the longest-running organised medical/PhD programme in Australasia is through the University of Otago in New Zealand [4]. Students interested in undertaking formalised research may decide to intercalate a BMedSc(Hons) or PhD at any stage up to the 5th year of the medical degree. Typically, most students complete the first three years of the six-year medical degree, then spend at least two research years, before going back to completing another three years of clinical medicine (i.e., 3 + 2 + 3 approach). Alternatively, students may elect to do the first five years of the medical degree, followed by at least two research years, before going back to complete the final year of the medical course (i.e., 5 + 2 + 1 approach) [5].

We have previously described the Otago MBChB/PhD student cohort (Alamri et al, *in press*). We found the matriculation rate to the MBChB/PhD programme to be comparatively low over the 18-year study period (an average of 1.4 students per year). For such an intercalated programme to be sustainable, problems need to be identified and resolved, and strengths ought to be recognised and augmented.

In order to explore this issue, we chose Vroom’s expectancy theory [6] as a theoretical framework. It was chosen as we anticipated motivations and perceived benefits of the effort of participation in the degree, for both students and supervisors, would be prominent, and that the theory would help us understand barriers to participation. Vroom’s expectancy theory explains motivation on the basis of expectancy (i.e., the extent to which a person believes the effort will result in a strong performance), instrumentality (i.e., the extent to which there is an association between performance and outcome), and valence (i.e., how much the person values the outcome).

The aim of the current study, therefore, was to explore the highlights, motivations and barriers of the intercalated programme (in its current format). These were obtained from both the students’ and supervisors’ perspectives.

## Methods

### Study setting and participants

As the total numbers of participants was expected to be small, we aimed to survey all students and supervisors of the MBChB/PhD programme (rather than needing to use any sampling or selection methods). A list of all MBChB/PhD students (past and current) and their supervisors was obtained from the Dean’s Office, Otago Medical School, Dunedin, New Zealand. Participants were invited to complete an online survey via email. Two additional reminder emails requesting participation were sent in four-week intervals. This study was approved by the University of Otago Human Ethics Committee (reference D18/019).

### Student and supervisor surveys

Draft questions were developed by the first author, and refined following discussions with the research team. As this was an exploratory study, the online surveys were designed as open-ended questions about problems encountered, programme strengths, advice for prospective students and supervisors, and suggestions for improvement. Participants were also encouraged to provide free-text comments about any other aspect of the MBChB/PhD programme not covered by the questions in an effort to capture the range of perceptions and experiences of the study participants. Finally, supervisors were also asked about their academic background, current position, and prior supervisory experience.

### Data analysis

Data were analysed using a general inductive approach which involved coding responses, and grouping codes into common themes via an iterative process. Two members of the research team coded the data independently; codes were then reviewed and revised by the whole research team. Finally, two researchers met face-to-face to finalise codes based upon consensus. Codes were grouped into themes by the research team as a whole. Responses to the student survey were considered separately from responses to the supervisor survey.

To explore student motivations and perceptions of an intercalated research degree, we used Vroom’s expectancy theory [6] as a theoretical framework. After the inductive coding was completed, and themes established, a deductive approach was used to relate the themes to the three variables of the theory (expectancy, instrumentality and valence).

## Results

### Study participants

Twenty-two past and current MBChB/PhD students responded to the survey; this gave a response rate of 88% (as 25 students had matriculated in the programme as of 2018). The characteristics and career outcomes of these students have been described elsewhere (Alamri et al, *in press*). In brief, of all MBChB/PhD matriculants, 15 completed both degrees; two students withdrew from the intercalated programme, and eight are still undertaking their research and/or medical degrees. Therefore, student responses ranged from experiences 14 years since graduation, to currently intercalating students. Of the 15 students who graduated, 12 have completed or are completing clinical speciality training, whilst three have taken research-focussed careers.

A total of 36 senior academics had supervised MBChB/PhD students, of whom 21 completed the survey (response rate 58.3%). All supervisors held one or more doctorate-level degrees—in the form of PhD (15/21), MD (7/21) and/or DSc (4/21). Of note, none of the supervisor-respondents were graduates of the MBChB/PhD programme. Seventeen of the respondents spent the majority of their work-time (> 80% full-time–equivalent) in research-related activities; three were full-time clinicians (estimated research-time 10–50% full-time–equivalent), and one was a retired professor. The median number of MBChB/PhD students supervised by each of the respondents was 1 (range, 1–5), without significant difference between research vs. clinician supervisors (*p* = 0.6). For respondents who supervised one MBChB/PhD student only (*n* = 13), the majority (9/13) acted as primary supervisors, rather than secondary (3/13) or tertiary (1/13) advisors. In addition, all respondents had supervised at least 8 post-graduate students at the time of the survey.

### Student responses

Three themes were identified through the analysis of the students’ responses. These were: motives for undertaking the intercalated degree, effect on career development, and perceived barriers.

#### Motives for undertaking the intercalated degree

Responses from students revealed two key motives for undertaking the MBChB/PhD degree. These were: gaining research skills and experience, and acquiring life skills and personal development.

##### Gaining research skills and experience

Most students cited gaining in-depth research skills as a main motive for undertaking the intercalated degree. Many thought that the PhD component offered research experiences above and beyond what a BMedSc(Hons) degree would offer. For some, the research experience transcended the confines of the laboratory to also positively influence their clinical careers.

“[Obtaining] research skills to a greater level than I would have received from a BMedSc(Hons)…” Student 16.

“… the [intercalated] programme has left me an engaged, research-informed clinician.” Student 6.

##### Acquiring life skills and personal development

A few of the students commented that they enrolled in the MBChB/PhD programme as a personal challenge. For others, the experience enabled personal development, and the accumulation of life skills applicable to, but outside of, academia.

“[To] test [the] limits of your ability…” Student 12.

“[The programme provided] an extra 2 years of growth/maturity before starting [clinical medicine].” Student 16.

“[I] learned how to work independently and self-motivate.” Student 18.

Applying the lens of Vroom’s theory, these themes are grossly consistent with the components of expectancy and instrumentality in that effort, or in this case the participation in the intercalated degree, is thought to lead to an outcome, in this case the acquisition of skills or personal development. Likewise, these themes reflect the variable of valence, in that the outcome in question is felt to hold value. Less apparent in students’ motivations, however, is an explicit reflection from students on performance, and the role that Vroom would suggest it plays in linking effort to outcome.

#### Effect on career development

The second theme, the effect of the MBChB/PhD programme on career development and progression, was central to the students’ experience. Two key subcategories were identified herein: the interplay between clinical medicine and research, and the influence of the programme on future careers.

##### Interplay between clinical medicine and research

Several of the students’ comments highlighted the complexity of the decision-making process related to intercalating a PhD with medical school training. Much of this complexity arose from the uncertainty about career paths and specialty choice, felt early in their medical school training. This contrasted the feeling from others that future obligations, such as family commitments, would make it difficult to complete postgraduate medical training, including rigorous clinical exams, separately and prior to undertaking a PhD.

“… you are early in your career, and may not be certain regarding your future speciality” Student 18.

“Many doctors choose to do their research degrees after their [post-graduate medical] exams, but as an older student with family commitments, I wouldn’t have been able to do this.” Student 10.

“[The MBChB/PhD is a] good path to follow if a clinical-research pathway is planned…” Student 22.

##### Influence of the programme on future careers

Comments from the students revealed the varied impact intercalation had on their career direction. This encompassed both the impact of the intercalated programme on career choice, including non-clinical career options, as well as the extent to which they have had continued involvement in research throughout their careers. The influence of the intercalated programme on the participant’s future careers varied from both positive to negative in quality.

“Indeed, (specific laboratory technique) influenced my choice of surgical training.” Student 8.

“[The programme] broadened my perspective on other careers, beyond medicine.” Student 22.

“I did not think I wanted to go into academia, and I was only trying the programme to get a feel of what research was like. However, I have really enjoyed it so far, and I would like to do [research] part-time in the future.” Student 16.

“[The programme] has not [influenced my career choice].” Student 14.

“I think I am now less likely to pursue a research career based on my experiences. [However], the research the work in (specialty) reinforced my decision to train in the area.” Student 6.

These themes, concordant with Vroom’s theory, reveal how the belief that one’s efforts will result in a desired performance is underpinned by self-efficacy and perceived control around goal attainment. Indeed, deciding to intercalate the PhD depended on personal attributes and objectives as well as perceived constraints, and the effect that these would have on effort, and therefore performance, and therefore successful outcome. Also revealed in these themes is that certain outcomes, including the effect of the intercalated programme on the participant’s future careers, were unanticipated at the outset. Accordingly, these outcomes were unlikely to have driven effort or performance, or to contribute to motivation, and therefore, fall out of the purview of Vroom’s theory.

### Perceived barriers

Multiple factors outside of their personal locus of control impacted the students’ experience. Key among them were administrative impediments, suboptimal support, and problematic phase transitions.

#### Administrative impediments

Several students identified the presence of administrative and logistical obstacles that made the intercalation journey more stressful. For example, some students noted that the intercalated research degrees (both BMedSc(Hons), and PhD) were not always well-advertised. For those who are undertaking a BMedSc(Hons), the option of upgrading to a PhD is not brought up (if at all) until very late. Even after successful intercalation, some students faced ongoing clerical difficulties.

“The way [the intercalated programme] was promoted seemed as though it was more for students who had already heard about it elsewhere, and knew to look out for it.” Student 1.

“Have [upgrading to PhD] as a clear pathway from BMedSc(Hons), and encourage those who are interested to start thinking about the larger scope project from early on.” Student 21.

“Better education of the doctoral office to make enrolment and the transition of BMedSc(Hons) to PhD process less stressful.” Student 22.

“If possible, have the Graduate School acknowledge any PhD work we do during [medical school]… [Without this], they don’t give us any of our [PhD] scholarship stipend” Student 18.

#### Support

Access to human (e.g., peers, informed supervisors and expert resources) and financial supports influenced the students’ experience, and their perceived success of the intercalated programme. Additionally, given the nuances of the MBChB/PhD programme, for example when compared with a “regular” PhD degree, it was felt that support avenues ought to be to be specific and fit-for-purpose.

“Provide means/forum to allow MBChB/PhD students to interact, catch-up, and exchange knowledge and experience. Provide more social support networks. Currently, I feel as though I am only a PhD student and have nothing to do with the [other medical students].” Student 1.

“It may be helpful to consider extending financial support (in the form of PhD scholarships) to include a fourth year for PhD students who have taken longer due to the nature of the intercalated degree.” Student 5.

“[Departments] need to be more supportive—perhaps needs to come from leadership. Funding for projects, supervision and expectations—these need to be different from a normal PhD” Student 11.

“Greater acknowledgement of the [intercalated] programme within academic departments to allow flexibility with scheduling (e.g., to attend conferences)” Student 3.

#### Problematic phase transitions

Some students faced difficulties during transition phases from being a pre-clinical medical student to a research student, and then on to being to a clinical medical student. Due to these transitions, students felt that the intercalated experience can be isolating. This, in turn, was thought to lead to psychological distress, especially in instances where the research project was not successful.

“[The intercalated programme needs] to have more integration with [District Health Boards] to allow research to begin in medical school, but potentially continue on into [post-graduate] years.” Student 3.

Effectively, these factors were impediments to success. While strictly they lie outside Vroom’s expectancy theory, they appeared to impact students’ perceptions of how performance might lead to a successful outcome, and thus can be seen as being related to instrumentality.

#### Supervisor responses

Through the analysis, two themes were identified from the supervisors’ survey. These were: characteristics of successful students, and optimising the intercalated programme.

### Characteristics of successful students

Several supervisors felt that students should possess certain characteristics thought to make them more likely to be successful in completing both degrees in the intercalated programme. These included: intrinsic motivation, time-commitment, emotional maturity, and realistic expectations.

“It depends on their motivation for doing the programme.” Supervisor 2.

“… I have seen students overwhelmed by the demands of laboratory work, especially when things were not going smoothly” Supervisor 4.

“If you do not have good time management skills, consider delaying the PhD until after your medical studies are complete.” Supervisor 9.

“[The unsuccessful candidate] was less emotionally mature than other doctoral thesis candidates at the time of commencing their research component.” Supervisor 11.

“Select the project and supervisor very carefully. Speak with previous concurrent-degree students to get a clear picture of what it entails” Supervisor 14.

This aligns with Vroom’s theory, which suggests there are conditions, including personal attributes and experiences, that affect expectancy, or the notion that effort yields performance.

### Optimising the intercalated programme

Several supervisors expressed frustration with several aspects of the intercalated programme. These included: restrictive time-frames for the PhD component, logistical inadequacies, and the need for more rigorous student, supervisor and project selection.

#### Restrictive PhD time-frames

This was noted to be one of the main reasons behind supervisors’ dissatisfaction with the intercalated programme. This is complicated by the fact that MBChB/PhD students usually have to return to their medical course as full-time students, whilst trying to fit in unfinished research duties when able.

“[There are] difficulties in achieving momentum due to the punctuated structure of the intercalated course for some students (as opposed to later entry, with uninterrupted, full-time work on the project possible). Supervisor 7.

“[There is a] fragmented research time (both in terms of conducting experiments and thesis write up). The idea that using semester and end-of-year breaks as effective research time is misleading.” Supervisor 14.

“Later entry (after 5th year), with continuous full-time enrolment, is likely more manageable for the student and supervisors.” Supervisor 6.

#### Logistical inadequacies

Some of the supervisors expressed concerns about the limited access to financial resources. Others were frustrated with problems with student enrolment procedures.

“[The students’] lack of time [is compounded by the] lack of financial support.” Supervisor 13.

“The University system of enrolment when the student is back in [the clinical component] of the MBChB degree is flawed, and all my students have had problems with enrolment.” Supervisor 3.

“Recognition that [intercalating] students are doing both programmes at the central administrative level of the University [would improve it]”. Supervisor 2.

#### The need for more rigorous student, supervisor and project selection

To ensure the success of the intercalated programme, some supervisors advocated for more careful selection of students, supervisors and the type of research projects. A few respondents recommended the inclusion of an unbiased mediator to resolve any potential conflicts.

“Beware that unless you already had [research] skills, your undergraduate medical course has likely not equipped you well for producing formal academic writing.” Supervisor 7.

“To make [academic staff] aware of the programme to aid students in selecting projects and supervisors.” Supervisor 19.

“Select a project that you and your supervisor think is compatible with the style of the programme.” Supervisor 5.

“Anything that would enable students to have a mentor, other than their supervisor, to whom they could turn would be useful.” Supervisor 3.

“[Students need to] get good advice from senior clinical researchers without conflict of interest in the research being done.” Supervisor 16.

This theme, akin to the perceived barriers identified by students, reflects impediments to success. Systems, logistics, and support appear to impact perceptions of how effort leads to success. Thus, this theme is separate from, but related to, the variables of Vroom’s theory.

## Discussion

In the present study, we have shed light on perceptions of students and supervisors of the longest-running MBChB/PhD programme in Australasia. Three themes emerged through the analysis of the students’ responses. These were: motives for undertaking the intercalated degree, effect on career development, and perceived barriers. Supervisors’ survey also yielded two themes: characteristics of successful students, and optimising the intercalated programme. Within these themes, some factors were common across the students and their supervisors.

We applied Vroom’s theory to the deductive interpretation of the themes. We found most responses from students and their supervisors revealed motivations consistent with Vroom’s expectancy theory. Equally, in addition to the variables of Vroom’s theory (expectancy, instrumentality, and valence), we identified an added element of significance for both students and supervisors, related to logistics and support. While separate from Vroom’s three variables, logistics and support appear to modulate perceptions of how effort translates to performance (expectancy), and how performance translates to outcomes (instrumentality), and may therefore impact motivation.

Consistent with Vroom’s theory, students’ motivations for undertaking the intercalated degree were underpinned by the view that participation would lead to outcomes, including the acquisition of skills and personal development, and that these outcomes have value. The perceived impact of undertaking the intercalated program on career development was influenced by personal attributes and objectives as well as perceived constraints. Consistent with Vroom’s theory, these factors can be understood as impacting effort, which affects performance, and in turn, affects outcomes. Likewise, when interpreted through the lens of Vroom’s theory, the characteristics of successful students—identified in the supervisors’ responses—may be significant because they moderate instrumentality, or, put another way, affect how effort yields performance.

Responses from students of the intercalated programme and their supervisors overlapped in two key areas. These were related to the interplay between medical and research careers, and logistical challenges. Some of the students appreciated the juxtaposition of research and clinical medicine at such an early stage in their careers—if the experience was not a motive for further research, it at least provided guidance on future specialty of choice. Other students recognised this opportunity as the only time for them to complete substantial research (due to, for example, anticipated future family commitments).

From the supervisors’ perspective, the repeated interruption of research-time (by resuming clinical medicine) was undesirable. In addition, a few supervisors believed that some of the intercalating students lacked maturity (academic and emotional), and would have preferred if these students completed their medical degrees first. What both groups agreed upon was the presence of multiple logistical challenges facing students and supervisors alike. As with most research endeavours, there is always a need for more funding. However, even with the current funding available, under-recognition of the nature of the intercalated programme meant that some students could not access their PhD stipend once they had gone back to the medical degree (despite still working on their PhD research). Both parties agreed that adequate clerical awareness and support are lower than desirable.

Several factors highlighted by our participants (both students and their supervisors) have been emphasised as areas in need of improvement by leaders of the physician-scientist training programmes in the US [7]. These include the necessity of balancing research and medical careers, the need for strong institutional support and financial funds, and the reliance on adequately trained mentors and supervisors [7].

Several limitations to the study ought to be mentioned. Responses were voluntary and retrospective in nature; thus, raising the possibility of selection and recall (e.g., post-graduate experience affecting the recollections of student-participants) biases. The qualitative data provided insight into the experiences of some, but not all, MBChB/PhD students and supervisors. However, the response rates were very high, considering the typical response rates in medical education research of _~_ 45% [8]. Using questionnaire data to answer qualitative questions is suboptimal. The free-text, electronic format may limit the content and quality of responses, and forces interpretation of responses without the ability to clarify ambiguity. Indeed, there is no capacity to probe responses to elicit intended meaning or to develop incomplete or unclear ideas. Furthermore, the data are limited by the questions posed, and therefore run the risk of reflecting the authors’ biases as developers of the questionnaire. The research team acknowledge their own biases as relative “insiders” with research and publication experience and professional connections to the University of Otago. Diversity of roles and perspectives within the research team (Professor, medical registrar, and house officer), however, ensured high inter-rater reliability, and the number of respondents allowed saturation within the data to be achieved.

## Conclusions

The current study sheds light on the successes and challenges of an intercalated MBChB/PhD programme by considering the views of those most involved—intercalating students and their supervisors. Whereas the combined programme has its advantages for student research and career development, extending the research-time may be worthwhile. Currently, the number of intercalating students in New Zealand is too low to allow for meaningful comparisons (e.g., entry after 3rd vs. after 5th years, or success rate of undergraduate-entry vs. postgraduate-entry). Further studies involving a larger cohort of intercalating students (e.g., combining Australia and New Zealand data), whilst sub-optimal due to the different settings, may allow for extrapolation of data to address these concerns.

## Data Availability

The datasets used and/or analysed during the current study are available from the corresponding author on reasonable request after completion of data publication.
